# Long-Term Impact of *Toxoplasma gondii* Infection on Human Monocytes

**DOI:** 10.3389/fcimb.2019.00235

**Published:** 2019-06-28

**Authors:** Hauke G. Ehmen, Carsten G. K. Lüder

**Affiliations:** Institute for Medical Microbiology, University Medical Center Goettingen, Georg-August-University, Goettingen, Germany

**Keywords:** toxoplasmosis, humans, monocytes, monocyte subsets, cytokines, surface markers, chronic infection

## Abstract

*Toxoplasma gondii* is a prevalent parasite of mammals and birds including up to 30% of humans world-wide. Primary infection of immunocompetent hosts leads to a robust cell-mediated immune response, which controls but does not clear the infection, thus enabling long-term parasite persistence in brain and muscle tissues. Chronic toxoplasmosis in mice is associated with resistance to heterologous pathogens and this has been related to increased numbers of inflammatory monocytes. Here we have analyzed whether chronic *T. gondii* infection impacts the subset distribution and the phenotype of peripheral human monocytes *in vivo* and their responses to parasite infection *in vitro*. CD14^+^ monocytes from *T. gondii*-seropositive blood donors expressed significantly less FcγRIII (CD16) than those from seronegative controls, but they did not show a shift in the distribution of classical, intermediate and non-classical monocyte subpopulations. Percentages of CD62L^+^ and CD64^+^ monocytes were however decreased and increased, respectively, in chronically infected individuals as compared to naïve controls. Infection of monocyte-enriched PBMCs from both seropositive and seronegative individuals with *T. gondii* led to an increase of CD14^+^CD16^−^ classical monocytes and a decrease of CD14^+^CD16^+^ double positive monocytes. Remarkably, after *in vitro* parasite infection, expression of the chemokine receptor CCR2 was severely impaired in monocytes from both, individuals with chronic toxoplasmosis and seronegative controls. In contrast, only monocytes from chronically infected humans but not those from controls dose-dependently up-regulated HLA-DR, DP, DQ expression following *in vitro* infection. Furthermore, monocyte-enriched PBMCs from seropositive individuals up-regulated IL-12 mRNA more vigorously after *in vitro* infection than cells from naïve controls. Collectively, our results establish that infection of humans with *T. gondii* exerts long-term effects on the phenotype and responsiveness of blood monocytes. This may have important implications for innate immune responses to *T. gondii* and unrelated pathogens.

## Introduction

The intracellular parasite *Toxoplasma gondii* is widespread in birds and mammals including an estimated 30% of humans world-wide. Infections of immunocompetent hosts are mostly asymptomatic or benign but they lead to parasite persistence for months to years and possibly even for the hosts' life. Reactivation of chronic toxoplasmosis in immunocompromised individuals, e.g., those with AIDS or under immunosuppressive therapy, can however lead to necrotizing tissue damage and life-threatening *Toxoplasma* encephalitis (Montoya and Liesenfeld, 2004). Furthermore, primary infection of pregnant women can lead to intrauterine transmission with severe symptoms or even death of the fetus or significant sequelae after birth. *T. gondii* is also an important cause of posterior uveitis after infection of otherwise healthy adolescents or adults (Pleyer et al., 2014). Together, human toxoplasmosis has been recognized as one of the leading food-borne infectious diseases in the USA based on annual costs and loss of quality-adjusted life years (Hoffmann et al., 2012).

After primary infection, infectious sporozoites or bradyzoites penetrate enterocytes and transform within cells of the lamina propria to metabolically highly active tachyzoites. Tachyzoites disseminate throughout the host by several rounds of fast replication within various host cells including monocytes and dendritic cells (DCs). These innate immune cells sense the parasite by Toll-like receptor (TLR)-dependent (Yarovinsky et al., 2005; Debierre-Grockiego et al., 2007; Andrade et al., 2013) and –independent mechanisms (Witola et al., 2011; Gov et al., 2013; Ewald et al., 2014; Gorfu et al., 2014) in a partially host species-dependent manner (Sher et al., 2017). They subsequently secrete interleukin (IL)-12 that is critical to stimulate early interferon (IFN)-γ production by natural killer (NK) cells (Suzuki et al., 1988; Gazzinelli et al., 1993, 1994). Priming and differentiation of CD4^+^ and CD8^+^ T lymphocytes further contribute to robust IFN-γ secretion (Suzuki and Remington, 1988; Gazzinelli et al., 1991) which in turn activates hematopoietic and non-hematopoietic cells (Yap and Sher, 1999) to exert various effector mechanisms in a host cell type- and host species-specific manner (Yarovinsky, 2014; Krishnamurthy et al., 2017). Importantly, some tachyzoites are able to evade killing by stage differentiation to latent and slowly to non-replicating bradyzoites predominantly within neurons and muscle cells. Bradyzoites rebuild the PV to an intracellular tissue cyst that is surrounded by a robust cyst wall and that marks the chronic phase of infection. The immune response to the bradyzoite stage is largely unexplored, but it is clear that IL-12 (Yap et al., 2000) and IFN-γ secretion by CD8^+^ and CD4^+^ T lymphocytes (Gazzinelli et al., 1992) are critical to control the chronic phase of infection. What remains unknown however is whether such parasite control is mediated by immune responses to the bradyzoite stage or rather by killing of tachyzoites which emerge after occasional re-differentiation of the latent stage and tissue cyst rupture.

Remarkably, mice infected with *T. gondii* are more resistant to infections with *Listeria monocytogenes* or *Salmonella enterica* ser. Typhimurium than non-infected controls (Ruskin and Remington, 1968; Neal and Knoll, 2014). Resistance against heterologous pathogens persists for several months and appears to be mediated by macrophages (Ruskin and Rengton, 1968; Ruskin et al., 1969). Mechanistic studies recently showed that Ly6C^+^ “inflammatory” monocytes which are recruited after stimulation of TLR11 by *T. gondii* profilin (TgPRF) in a CCR2-dependent manner are able to confer resistance against bacterial infection in mice (Neal and Knoll, 2014). Ly6C^+^ monocytes are recruited to the site of infection during infections with several intracellular pathogens including *L. monocytogenes* and *T. gondii* (Robben et al., 2005; Serbina and Pamer, 2006) and are critical to control these pathogens (Dunay et al., 2008; Serbina et al., 2012), presumably by directly exerting antimicrobial effector mechanisms. TgPRF is a soluble protein functioning in actin remodeling in *T. gondii* (Plattner et al., 2008). It is a major IL-12 inducer after being bound to TLR11/TLR12 on DCs from mice (Yarovinsky et al., 2005; Plattner et al., 2008) but not humans who lack these receptors. Together, these results suggest that in *T. gondii*-infected mice distinct monocyte populations can confer lasting resistance to heterologous infections.

In humans, three distinct monocyte subpopulations have been described based on their expression of CD14 and CD16 (Ziegler-Heitbrock et al., 2010). The so-called classical monocytes account for ~85% of the circulating monocyte pool under steady state conditions, they are CD14^+^ and CD16^−^, and appear to be the human counterparts of Ly6C^+^CD43^low^CCR2^high^ monocytes from mice (Ziegler-Heitbrock et al., 2010). The remaining human monocytes are the non-classical ones which are CD14^dim^CD16^+^ and those with an intermediate phenotype (CD14^+^CD16^+^). Additional surface markers have been proposed to distinguish between these monocyte subsets (Ingersoll et al., 2010; Ziegler-Heitbrock et al., 2010; Patel et al., 2017). Of note, after emigration of classical monocytes from the bone marrow in a CCR2-dependent manner, they appear to sequentially develop into intermediate and then non-classical monocytes, indicating that monocytes are quite dynamic (Patel et al., 2017) and subset boundaries not always easy to define (Ziegler-Heitbrock et al., 2010). Importantly, the distribution of monocyte subsets and their functional properties change during distinct pathologies including infectious and non-infectious inflammatory diseases (Fingerle et al., 1993; Fingerle-Rowson et al., 1998; Horelt et al., 2002; Patel et al., 2017).

Here, we have analyzed the subset distribution of peripheral monocytes and their phenotypic and functional properties from chronic toxoplasmosis patients and from naïve control individuals. Results for the first time indicate differences in the repertoire of monocyte surface markers expressed during human chronic toxoplasmosis but not a shift in the distribution of the three major subsets as compared to control individuals. *In vitro* infection of monocyte-enriched PBMCs led to an expansion of the classical monocyte subset from both chronically infected and naïve individuals, and to an up-regulation of HLA-DR,DP,DQ, and a more vigorous IL-12 response specifically in cells from chronic toxoplasmosis patients. Long-term effects of *T. gondii* infection on innate immune cells of humans can have important consequences on their reactivity to homologous and heterologous pathogens.

## Materials and Methods

### Study Population and Ethics

Buffy coats and plasma from heparinized blood of healthy volunteers were obtained from the Blood Donation Service Center of the University Medical School Göttingen, Germany. Study subjects were excluded from donating blood when presenting pathologies indicative for transmissible infectious diseases, being at risk of having blood products-transmitted infectious diseases, or presenting severe non-infectious diseases which precluded regular blood donation. All donors gave written informed consent, and the study was approved by the Ethics commission of the University Medical School Göttingen (Project numbers 28/3/08 and 1/3/13). Plasma was obtained by centrifugation of blood aliquots at 3,000 × *g* for 5 min. Serological tests on plasma samples for detection of *T. gondii*-specific total immunoglobulins, *T. gondii*-specific IgG, IgM and IgA and for measuring the avidity of anti-*T. gondii*-specific IgG were performed at the Institute for Medical Microbiology (Göttingen, Germany) using standardized routine procedures.

### Isolation of PBMCs and Enrichment of Monocytes

Peripheral blood mononuclear cells (PBMCs) were isolated by Ficoll-Paque Plus (GE Healthcare Life Sciences, Freiburg, Germany) density centrifugation of blood diluted in RPMI 1640 medium at 900 × *g* for 30 min. They were extensively washed and monocytes were then enriched by allowing them to adhere to polysterene cell culture dishes (Greiner Bio-One, Frickenhausen, Germany) in RPMI 1640 medium, 100 U/ml penicillin and 100 μg/ml streptomycin for 2 h at 37°C and subsequent removal of non-adherent cells. The monocyte-enriched PBMCs were then directly analyzed by fluorescence-activated cell sorting (FACS), or they were incubated in RPMI 1640, 10% heat-inactivated fetal calf serum and antibiotics as above for infection assays at 24 h after isolation.

### *T. gondii* and Parasite Infection

The mouse-avirulent *T. gondii* type II strain NTE (Gross et al., 1991) was used throughout this study. Tachyzoites were propagated in L929 fibroblasts as host cells. For infection assays, freshly egressed parasites were separated from host cells by differential centrifugation as described previously (Lang et al., 2006). After having been extensively washed, they were added to monocyte-enriched PBMCs at multiplicities of infection (MOI) of 3:1 and 6:1 for 24 h.

### Flow Cytometry

Expression of cell surface markers by human monocytes was quantified by FACS. To this end, freshly isolated monocyte-enriched PBMCs or those cultivated and/or infected with *T. gondii in vitro* were detached from tissue culture dishes using cell scrapers and were washed twice in PBS, pH 7.4. For each staining, 500,000 cells were transferred into the wells of a 96-well-V-bottom microplate and unspecific binding sites were blocked with 1% human AB serum, 1% bovine serum albumin (BSA), 0.1% NaN_3_ in PBS, pH 7.4 during 30 min at 4°C. Cells were then co-stained for 30 min at 4°C using FITC-conjugated mouse anti-human CD14 (clone M5E2) and PE-conjugated mouse anti-human CD16 (clone 3G8) or appropriate isotype control antibodies (clones G155-178 and MOPC-21; all antibodies from BD Biosciences, Heidelberg, Germany; diluted at 1:5 in 1% BSA, 0.1% NaN_3_ in PBS). Alternatively, they were labeled with 2 μg/mL of mouse monoclonal antibodies directed against human CD62L (clone DREG-56), human CD64 (clone 10.01.13), human HLA-A,B,C (clone G46-2.6), human HLA-DR,DP,DQ (clone Tu39; all antibodies from BD Biosciences), or directed against human CCR2 (R&D Systems, Wiesbaden-Nordenstadt, Germany), or they were incubated with appropriate isotype control antibodies (clones MOPC-21, 27-35 and G155-178; BD Biosciences) for 30 min at 4°C. After having been washed three times in 1% BSA, 0.1% NaN_3_ in PBS, pH 7.4, immune complexes were labeled with R-PE-conjugated goat F(ab')_2_ fragment anti-mouse IgG (diluted at 1:50 in 1% BSA, 0.1% NaN_3_ in PBS for 30 min at 4°C). After immunolabeling, cells were washed (as above) and were then fixed using 1% paraformaldehyde in PBS, pH 7.4. Ten thousand cells per sample were analyzed using a FACSCalibur (BD Biosciences).

### RNA Isolation and Quantitative Reverse Transcriptase-PCR

Total RNA was isolated from monocyte-enriched PBMCs after their isolation or after cultivation and/or infection with *T. gondii in vitro*, using the GenElute Mammalian Total RNA Miniprep kit (Sigma-Aldrich, Taufkirchen, Germany). Contaminating genomic DNA was digested with DNase I (amplification grade; Sigma-Aldrich) as recommended by the manufacturer. After reverse transcription of mRNA using the Omniscript RT kit (Qiagen, Hilden, Germany) and oligo(dT) primers, cDNAs were amplified by LightCycler quantitative PCR using the LightCycler FastStart DNA Master^PLUS^ SYBR Green I kit as recommended (Roche, Mannheim, Germany). Human transcripts were amplified using primer pairs specific for *il-12b* (p40) (forward: 5′-ATGCCCCTGGAGAAATGGTG-3′; reverse: 5′-GAACCTCGCCTCCTTTGTGA-3′), *il-10* (forward: 5′- GGCGCTGTCATCGATTTCTTC−3′; reverse: 5′- TAGAGTCGCCACCCTGATGT−3′), *tnfa* (forward: 5′- GCCCATGTTGTAGCAAACCC−3′; reverse: 5′- GGAGGTTGACCTTGGTCTGG−3′) and the reference gene β*-actin* (forward: 5′- TATCCAGGCTGTGCTATCCC−3′; reverse: 5′- CCATCTCTTGCTCGAAGTCC−3′). The relative gene expression levels were calculated as the fold changes between freshly isolated (0 h) and *in vitro*-cultivated, non-infected cells (48 h, non-infected) or between freshly isolated (0 h) and *T. gondii*-infected cells (48 h, *T. gondii*) using the formula Ratio = ${\left(E_{\text{target}}\right)}^{\text{Δ}CPtarget\left(0hours-48hours/\pm T.\;gondii\right)}$/${\left(E_{\text{ref}}\right)}^{\text{Δ}CPref\left(0hours-48hours/\pm T.\;gondii\right)}$, where E denotes real-time PCR efficiencies and ΔCP the crossing point (CP) differences (Pfaffl et al., 2002).

### Statistical Analyses

Outlyers within datasets were identified by Dixon's Q-test and were not further considered. Data are presented as means ± S.E.M. or as box-whisker plots indicating the mean, median and percentiles as indicated. Individual data points have also been included within graphs. Significant differences were identified by Student's *t*-test or by ANOVA of between groups and repeated measures matrices using the General ANOVA/MANOVA module of Statistica (Version 13.3.; TIBCO Software, Palo Alto, CA, USA). *P*-values of <0.05 were considered significant.

## Results

### Study Subjects

Buffy coats from 21 healthy blood donors were included into this study. Screening of plasma from blood samples using the VIDAS® *Toxoplasma* IgG/IgM competition kit (bioMerieux, Nürtingen, Germany) identified specific antibodies in 5 out of 21 individuals (23.8%; Supplementary Table 1). Subsequent differentiation of the positive samples revealed *Toxoplasma*-specific IgG in all samples (VIDAS® TOXO IgG II; 24–>300 IU/ml), *Toxoplasma*-specific IgM in two of them (VIDAS® TOXO IgM; index values 0.94 or 1.95), and no specific IgA in any sample (Platelia Toxo IgA (TMB); BioRad, München, Germany). Furthermore, high avidities were found for *Toxoplasma* IgG antibodies from the two IgM-positive samples (VIDAS® TOXO IgG Avidity; Supplementary Table 1). Serological data thus indicated chronic (> 4 months) *T. gondii* infections in 5 blood donors whereas no signs of infection were found in 16 controls.

### Expression of Surface Markers Differs Between Monocytes From Chronic Toxoplasmosis Patients and Naïve Controls

**Figure 1:**
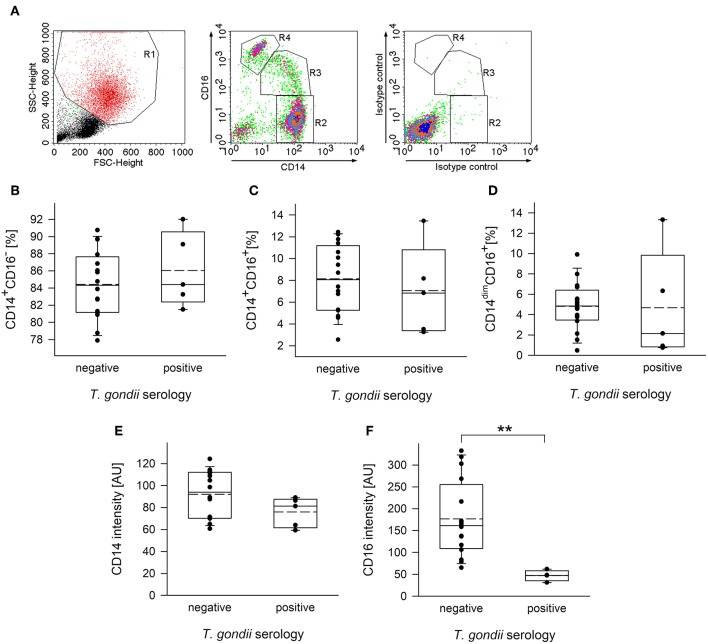
Distribution of subpopulations and expression levels of CD14 and CD16 of monocytes from blood donors with or without chronic toxoplasmosis. PBMCs were isolated from blood by density centrifugation and were enriched for plastic-adherent monocytes. They were then stained using fluorescently labeled anti-CD14 and anti-CD16 reagents or isotype control antibodies and were FACS-analyzed. Blood donors were serologically classified as chronically *T. gondii*-infected or non-infected using plasma from the blood samples. **(A)** Representative FACS analysis of monocyte-enriched PBMCs from a *T. gondii* naïve blood donor showing FSC/SSC-based gating on monocytes (left panel) and CD14/CD16-based identification of classical (R2; i.e., CD14^+^CD16^−^), intermediate (R3; i.e., CD14^+^CD16^+^) and non-classical (R4; i.e., CD14^dim^CD16^+^) monocyte subsets. Staining with isotype control antibodies is shown in the right panel. **(B–D)** Percentages of CD14^+^CD16^−^ **(B)**, CD14^+^CD16^+^ **(C)**, and CD14^dim^CD16^+^ **(D)** subsets among monocytes from *T. gondii* seropositive or seronegative individuals. Solid and dashed lines in the box-whisker plots indicate median and mean values, respectively; circles indicate individual data points. **(E,F)** Expression levels of CD14 **(E)** and CD16 **(F)** on CD14^+/dim^ monocytes, i.e., cells within R2+R3+R4 from *T. gondii* seropositive or seronegative blood donors. Data are from 5 *T. gondii* seropositive and 16 seronegative blood donors; outlyers were excluded. Significant differences between both groups were identified by Student's *t*-test (^**^p < 0.01).

Monocytes from human peripheral blood are classified into three subpopulations based on surface expression of CD14, i.e., the co-receptor for lipopolysaccharide (LPS), and CD16, i.e., the type III Fcγ receptor (FcγRIII) (Ziegler-Heitbrock et al., 2010). In order to distinguish between these subsets, plastic-adherent PBMCs were fluorescently labeled using FITC-conjugated anti-CD14 and PE-conjugated anti-CD16 reagents or appropriate isotype control antibodies and analyzed by FACS. Results confirmed three monocyte subsets, i.e., CD14^+^CD16^−^ classical monocytes (R2 in Figure 1A), CD14^+^CD16^+^ intermediate monocytes (R3) and CD14^dim^CD16^+^ non-classical monocytes (R4) among the PMBCs from chronically infected toxoplasmosis patients and non-infected controls (Figures 1A–D). In contrast, isotype control antibodies did not specifically bind to these cells (Figure 1A). CD14^+^CD16^−^ monocytes clearly predominated, with ~85% of the total CD14^+^ cells from *T. gondii* seronegative and seropositive individuals belonging to this subset (Figure 1B). CD14^+^CD16^+^ intermediate monocytes accounted for 7.0 to 8.1% (means) and CD14^dim^CD16^+^ non-classical monocytes for ~4.75% of the total CD14^+^ cells and this did not grossly differ between *T. gondii*-infected and non-infected individuals (Figures 1C,D). CD14^+/dim^ monocytes were further analyzed for expression levels of CD16 and CD14 (see Supplementary Figure 1 for a representative FACS analysis). Remarkably, expression levels of CD16 were significantly lower on monocytes from chronically infected toxoplasmosis patients than those from naïve controls (*p* = 0.009, Student's *t*-test; Figure 1F). We also recognized a trend toward lower CD14 levels on monocytes from *T. gondii*-positive individuals as compared to controls, but this did not reach statistical significance (*p* = 0.133; Figure 1E).

**Figure 2:**
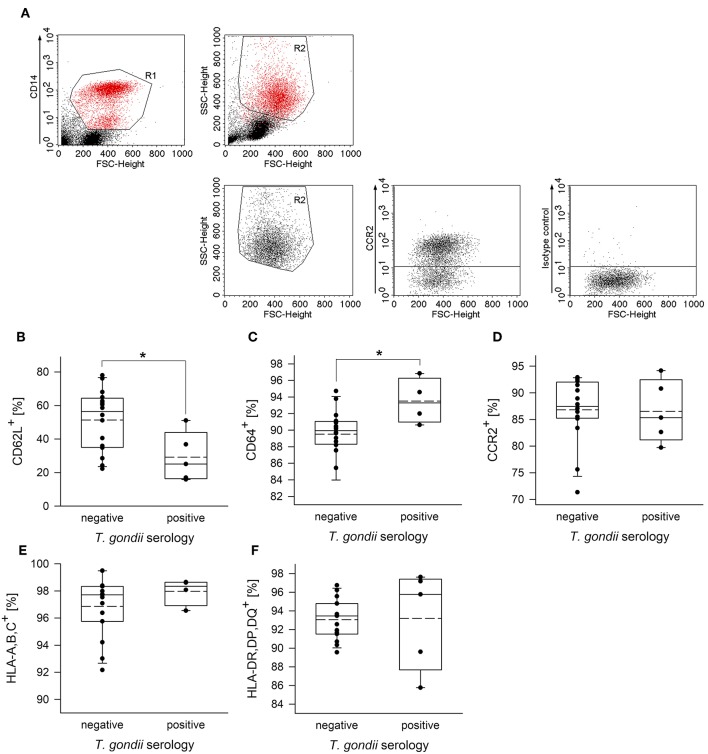
Proportion of CD62L^+^, CD64^+^, CCR2^+^, HLA,A,B,C^+^ or HLA,DR,DP,DQ^+^ cells among peripheral blood monocytes from donors with or without chronic toxoplasmosis. Plastic-adherent PBMCs were isolated from blood samples and were fluorescently labeled using FITC-conjugated anti-CD14 or antibodies directed against the indicated surface markers or isotype control antibodies and PE-conjugated secondary antibodies. Blood donors were serologically classified as chronically *T. gondii*-infected or non-infected using plasma from the blood samples. **(A)** CD14^+^ monocytes (R1) were back-gated and identified (R2) among FSC/SSC-analyzed total cells. R2-gated cells were then analyzed for expression of cell surface markers as indicated, and positive cells were identified after specific (anti-CCR2 in **(A)**; see Supplementary Figure 2 for the other surface markers) and isotype control labeling. **(B–F)** Percentages of monocytes from *T. gondii* seropositive or seronegative individuals positive for cell surface markers as indicated. Solid and dashed lines in the box-whisker plots indicate median and mean values, respectively; circles indicate individual data points. Data are from 5 *T. gondii* seropositive and 16 seronegative blood donors; outlyers were excluded.^*^p < 0.05 (Student's *t*-test).

To further unravel phenotypic differences between monocytes from chronically infected toxoplasmosis patients and non-infected controls, we stained freshly isolated plastic-adherent PBMCs also for other cell surface proteins which have been considered as additional markers of monocyte subsets (Ingersoll et al., 2010; Ziegler-Heitbrock et al., 2010; Patel et al., 2017) or which are crucial for cellular functions. To this end, CD14^+^ and CD14^dim^ cells were backgated onto FSC vs. SSC to identify the monocyte population, and these cells were then analyzed for expression of L-selectin (CD62L), high affinity FcγRI (CD64), chemokine receptor CCR2 (CD192), MHC class I (HLA-A,B,C) and class II (HLA-DR,DP,DQ) (see Figure 2A and Supplementary Figure 2 for representative examples of the gating strategy and labeling of different surface markers, respectively). Staining of the cells with isotype control antibodies were used to set thresholds for positive cells whereas expression levels were determined for R2-gated cells (Figure 2A and Supplementary Figure 2). Results revealed significantly less CD62L^+^ monocytes in chronically infected toxoplasmosis patients as compared to naïve controls (Figure 2B). Expression levels of CD62L were also considerably lower on monocytes from seropositive individuals than on those from control individuals although this did not reach statistical significance (Table 1). CD62L is involved in adhesion of leukocytes to endothelial cells and facilitates early stages of emigration from the circulating blood toward the extravascular tissue (Rzeniewicz et al., 2015). It is more prevalent on mouse Ly6C^+^ and human CD16^−^ monocytes as compared to the respective Ly6C^low^ and CD16^+^ counterparts (Ingersoll et al., 2010). Mean expression levels of CCR2, i.e., a surface marker of CD14^+^CD16^−^ cells (Ingersoll et al., 2010) did not significantly differ between monocytes from seropositive individuals and those from seronegative controls (Table 1). Likewise, the proportion of CCR2^+^ cells was similar among monocytes from both groups (Figure 2D). CD64^+^ cells were to a small extent but significantly expanded within the monocyte population of patients with chronic toxoplasmosis as compared to that of seronegative controls (Figure 2C), but expression levels did not differ between both groups (Table 1). CD64 confers strong phagocytic activity and is mainly expressed on CD14^+^CD16^−^ classical and CD14^+^CD16^+^ intermediate but not on CD14^dim^CD16^+^ non-classical monocytes (Grage-Griebenow et al., 2001; Ingersoll et al., 2010). Finally, monocytes from both *T. gondii* seropositive and seronegative individuals did not differ with respect to percentages or expression levels of HLA-A,B,C and HLA-DR,DP,DQ (Figures 2E,F; Table 1). Together, these results establish that monocytes of chronically infected toxoplasmosis patients differ from those of control individuals by surface expression or positivity of CD16, CD62L, and CD64, but that the distribution of the three major monocyte subsets was not altered.

**Table 1:** Expression of cell surface receptors on monocytes from individuals with chronic toxoplasmosis and non-infected controls.

	**Non-infected**	**Chronic toxoplasmosis**	***P*-value**
CD62L	90.03 ± 11.6^*^	47.98 ± 10.2	*p* = 0.069
CD64	155.83 ± 14.05	146.9 ± 27.57	*p* = 0.765
CCR2	200.21 ± 31.06	146.17 ± 40.87	*p* = 0.383
HLA-A,B,C	1305.95 ± 100.05	1156.63 ± 317.58	*p* = 0.51
HLA-DR,DP,DQ	857.66 ± 73.4	895.2 ± 172.84	*p* = 0.817

* *Data are means ± S.E.M*.

### *Ex vivo* Infection With *T. gondii* Strongly Alters Monocyte Subpopulations and Expression of Surface Markers

**Figure 3:**
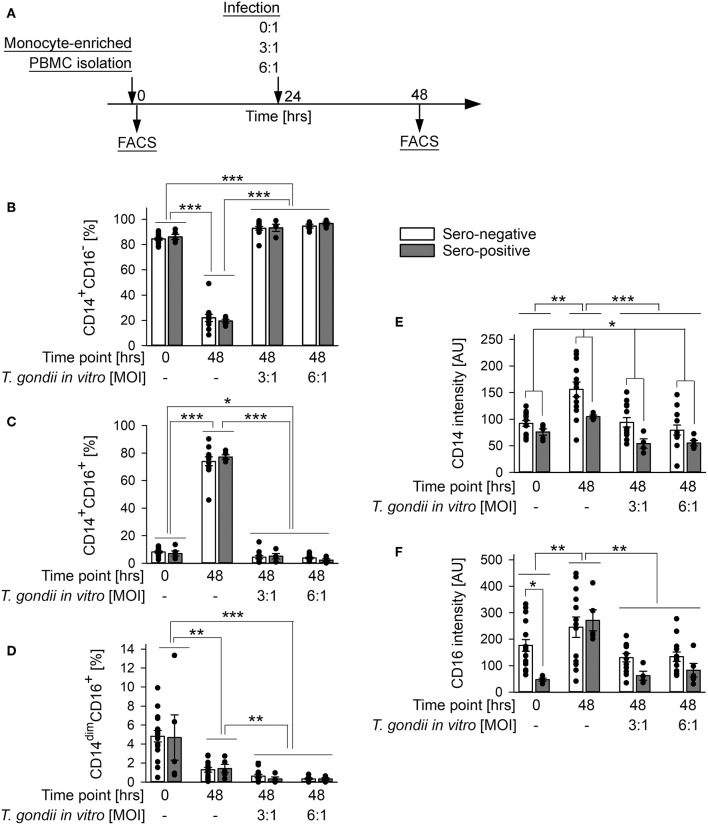
Impact of *in vitro* infection of monocyte-enriched PBMCs with *T. gondii* on subset distribution and expression of CD14 and CD16 of monocytes from blood donors with or without chronic toxoplasmosis. **(A)** Monocyte-enriched PBMCs were isolated from blood samples and were either directly FACS-analyzed (0 h) or cultivated *in vitro* for 48 h and parasite-infected or not during the final 24 h as indicated and then FACS-analyzed. **(B–D)** Percentages of CD14^+^CD16^−^ **(B)**, CD14^+^CD16^+^ **(C)** and CD14^dim^CD16^+^ **(D)** subsets among monocytes from *T. gondii* seropositive (gray bars) or seronegative (open bars) individuals. **(E,F)** Expression levels of CD14 **(E)** and CD16 **(F)** on monocytes from *T. gondii* seropositive or seronegative blood donors. Data represent means ± S.E.M. from 5 *T. gondii* seropositive and from 13 out of 16 seronegative blood donors which had been randomly selected for *in vitro* infection assays; outlyers were excluded. Individual data points are also indicated. Significant differences between groups were identified by ANOVA (^***^p < 0.001; ^**^p < 0.01; ^*^p < 0.05).

Monocyte subsets fulfill distinct functions during steady state and pathological conditions (Grage-Griebenow et al., 2001; Auffray et al., 2009; Ziegler-Heitbrock, 2015). We therefore wondered how human primary monocytes respond to direct exposure to *T. gondii* and whether reactivity to infection differs between monocytes from individuals who were previously exposed to the parasite or not. To this end, monocyte-enriched PBMCs were incubated *in vitro* for 48 h and were infected with *T. gondii* during the final 24 h or left non-infected (Figure 3A). They were then labeled with FITC-conjugated anti-CD14 and PE-conjugated anti-CD16 and FACS-analyzed as above. Infection with *T. gondii* led to a dramatic expansion of CD14^+^CD16^−^ classical monocytes (*p* < 0.001; ANOVA) with a simultaneous strong decrease of CD14^+^CD16^+^ intermediate (*p* < 0.001) and a moderate decrease of CD14^dim^CD16^+^ non-classical monocytes (*p* < 0.01), as compared to non-infected controls (Figures 3B–D). Importantly, the changes in subset distribution did not differ between monocytes from chronically infected toxoplasmosis patients and naïve controls, indicating a general response to the parasite. Furthermore, the parasite dose had no impact on the level of subset redistribution, since MOIs of 3:1 and 6:1 yielded almost identical results. An expansion of CD14^+^CD16^−^ monocytes (*p* < 0.001) and a concomitant reduction of CD14^+^CD16^+^ and CD14^dim^CD16^+^ (*p* < 0.05 and *p* < 0.001, respectively) monocytes after infection became also evident when results were compared to cells freshly isolated from the buffy coats (i.e., at time point 0 in Figures 3B–D). With respect to CD14^+^CD16^−^ and CD14^+^CD16^+^ subsets, these changes were however much lower when compared to those between non-infected controls and infected cells at 48 h of infection, since the mere *in vitro* incubation of monocytes for 2 days in the absence of *T. gondii* significantly impacted subset distribution (Figures 3B–D). It is interesting to note, that the changes in CD14^dim^CD16^+^ non-classical monocytes between freshly isolated cells and parasite-infected cells by contrast even exceeded those observed between non-infected monocytes at 0 and 48 h (Figure 3D). Consistent with changes in subset distribution, expression levels of CD14 and CD16 were also altered in response to the parasite, with both markers being decreased as compared to non-infected controls (*p* < 0.001 or *p* < 0.01, respectively; ANOVA; Figures 3E,F). This response abolished the increase in CD14 and CD16 expression as observed after *in vitro* incubation for 48 h without *T. gondii* (*p* < 0.01). Again, CD14 and CD16 surface expression were similarly modulated in monocytes from both, chronic toxoplasmosis patients and from naïve controls. Of note however, those from *T. gondii* seropositive individuals generally expressed less CD14 than those from control individuals (*p* < 0.05; ANOVA; Figure 3E), thus confirming a trend that we already recognized in freshly isolated cells (see above and Figure 1E). In contrast, CD16 levels were only significantly lower on monocytes from chronically infected toxoplasmosis patients directly after isolation (time point 0 in Figure 3F; *p* < 0.05; also see Figure 1F).

**Figure 4:**
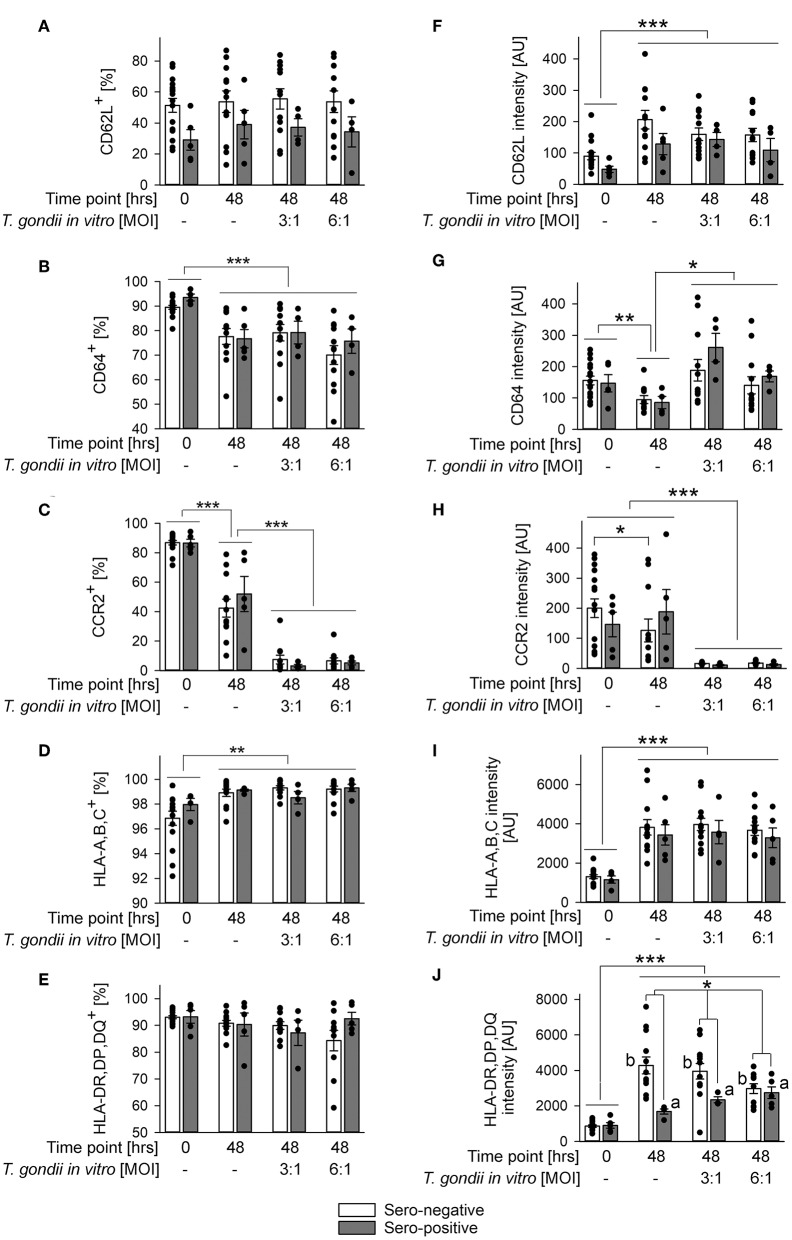
Impact of *in vitro* infection of monocyte-enriched PBMCs with *T. gondii* on expression of CD62L, CD64, CCR2, HLA-A,B,C, or HLA-DR,DP,DQ on monocytes from donors with or without chronic toxoplasmosis. Monocyte-enriched PBMCs were isolated from blood samples and were either directly FACS-analyzed (0 h) or were cultivated *in vitro* for 48 h and infected or not with *T. gondii* during the final 24 h as indicated and then FACS-analyzed. Expression of cell surface markers was determined for CD14-positive monocytes as outlined in Figure 2. **(A–E)** Percentages of cells from *T. gondii* seropositive (gray bars) or seronegative (open bars) individuals with expression of surface markers above background staining. **(F–J)** Expression levels of surface markers as indicated on monocytes from *T. gondii* seropositive or seronegative blood donors. Data represent means ± S.E.M. from 5 *T. gondii* seropositive and from 13 out of 16 seronegative blood donors which had been randomly selected for *in vitro* infection assays; outlyers were excluded. Individual data points are also indicated. Significant differences between groups were identified by ANOVA [^***^p < 0.001; ^**^p < 0.01; ^*^p < 0.05; a and b indicate dose-dependent increase or decrease, respectively, of HLA-DR,DP,DQ after parasite infection (*p* < 0.01)].

The impact of *in vitro* infection with *T. gondii* was further examined by also immunolabeling other surface proteins as above and by applying a gating strategy as indicated in Figure 2A. Parasite infection did not alter percentages of CD62L^+^ monocytes or their CD62L expression levels from both, chronically infected toxoplasmosis patients and naïve controls as compared to monocytes incubated without *T. gondii* (Figures 4A,F). CD62L expression levels however increased on cells from both groups during *ex vivo* cultivation for 48 h irrespective of being parasite-infected or not (Figure 4F; *p* < 0.001; ANOVA). Furthermore, monocytes from *T. gondii* seropositive individuals consistently showed a trend toward reduced percentages of CD62L^+^ and lower levels of CD62L than those from seronegative controls although this did not reach statistical significance (Figures 4A,F). This extended results as observed with cells directly labeled after isolation (Figure 2B, Table 1). Expression levels of CD64 increased during *in vitro* infection of monocytes with *T. gondii* as compared to non-infected controls (*p* < 0.05; ANOVA), and this did not significantly differ between cells from chronic toxoplasmosis patients and naïve individuals (Figure 4G). Such increase did however not lead to a change in the proportion of CD64^+^ monocytes after infection as compared to non-infected controls (Figure 4B). The proportion of CD64^+^ cells after parasite infection *in vitro* instead even decreased among monocytes from both groups of individuals when compared to freshly isolated cells (i.e., those analyzed at 0 h) but this was not specific to parasite infection since it also occurred by *in vitro* cultivation for 48 h in the absence of *T. gondii* (Figure 4B; *p* < 0.001; ANOVA). In sharp contrast, expression of the chemokine receptor CCR2 and the percentages of CCR2^+^ monocytes strongly diminished during *in vitro* infection when compared to non-infected control cells irrespective of whether monocytes originated from *T. gondii* seropositive or seronegative individuals (Figures 4C,H; *p* < 0.001; ANOVA). HLA-A,B,C was consistently up-regulated and the proportion of HLA-A,B,C^+^ cells increased during 48 h of *in vitro* cultivation (Figures 4D,I; *p* < 0.001 or *p* < 0.01, respectively; ANOVA). These changes were however similar on monocytes infected *in vitro* with *T. gondii* and non-infected control cells, and it did also not differ between monocytes from chronically infected individuals or non-infected controls (Figures 4D,I). Finally, whereas percentages of HLA-DR,DP,DQ^+^ monocytes did not significantly differ between monocytes from both groups of individuals or after *in vitro* parasite infection (Figure 4E), expression levels of these molecules clearly increased from 0 to 48 h of *in vitro* cultivation of cells and parasite infection (*p* < 0.001; ANOVA; Figure 4J). Furthermore, monocytes from both groups differed markedly in their responses to parasite infection; whereas those from chronically infected toxoplasmosis patients dose-dependently up-regulated HLA-DR,DP,DQ as compared to non-infected control cells, those from naïve control individuals dose-dependently down-regulated such expression (Figure 4J; *p* < 0.01). Consequently, monocytes from both groups of blood donors differed significantly in HLA-DR,DP,DQ expression following *in vitro* cultivation and parasite infection (*p* < 0.05). Together, the results revealed a dramatic expansion of CD14^+^CD16^−^ monocytes after parasite infection *in vitro* with a concomitant increase of CD64 expression but a strong decrease of CCR2. Whereas, these changes occurred irrespective of a chronic *T. gondii* infection of the blood donors, we also unraveled distinct differences in the *in vitro* reactivity of monocytes from chronically infected toxoplasmosis patients and *T. gondii* naïve individuals with respect to CD14 and HLA-DR,DP,DQ. It has to be stressed that *in vitro* cultivation of monocytes in the absence of *T. gondii* significantly impacted their phenotypes (Figures 3, 4). Depending on the surface marker under investigation, direct exposure to the parasite reversed (Figures 3B,C,E,F, and Figure 4G), augmented Figure 3D, and Figures 4C,H or did not alter (Figures 4B,D,F,I) these changes.

### Monocyte-Enriched PBMCs From Chronic Toxoplasmosis Patients Express High IL-12b mRNA Levels After *in vitro* Parasite Infection

**Figure 5:**
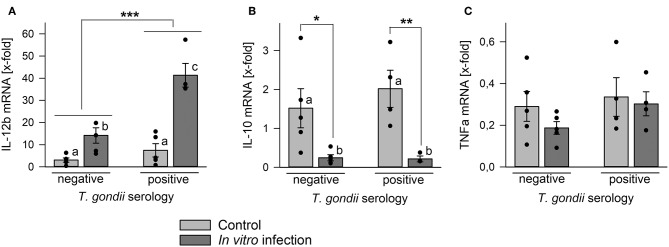
Regulation of mRNA levels of IL-12b, IL-10, and TNFa in monocyte-enriched PBMCs from blood donors with or without chronic toxoplasmosis after *in vitro* infection with *T. gondii*. Monocyte-enriched PBMCs were isolated from blood samples and blood donors were serologically classified as chronically *T. gondii*-infected or non-infected using blood plasma. Cells were either directly extracted for RNA isolation or they were cultivated for 48 h and parasite-infected (dark gray bars) or not (light gray bars) during the final 24 h. After RNA isolation, mRNA was reverse transcribed and cDNA amplified by quantitative real-time PCR using primer pairs specific to IL-12b **(A)**, IL-10 **(B)**, TNFa **(C)**, and ß-actin. Cytokine mRNA levels were normalized to ß-actin mRNA, and the regulation was calculated between cells directly isolated after blood donation and those after *in vitro* infection with *T. gondii* (dark gray bars) or after control cultivation (light gray bars). Bars represent means ± S.E.M. from 5 *T. gondii* seropositive and seronegative blood donors each; outlyers were excluded. Individual data points are also indicated. Significant differences between groups were identified by ANOVA (^***^p < 0.001; ^**^p < 0.01; ^*^p < 0.05); bars labeled with different letters differ significantly.

Monocytes are an important source for various cytokines under steady state conditions (Auffray et al., 2009) and in response to microorganisms including *T. gondii* (Yarovinsky, 2014; Sher et al., 2017). We therefore compared cytokine mRNA levels in monocyte-enriched PBMCs from chronic toxoplasmosis patients with those from naïve controls following *in vitro* infection with *T. gondii* or in non-infected control cells. Remarkably, cells isolated from *T. gondii* seropositive individuals up-regulated IL-12b (i.e., IL-12p40) mRNA significantly stronger in response to *in vitro* infection than those from seronegative control individuals (*p* < 0.001; ANOVA; Figure 5A). Relative IL-12b mRNA production was also slightly higher in cells from chronic toxoplasmosis patients than those from controls when cultivated for 48 h in the absence of the parasite but this was not statistically significant. Up-regulation of IL-12b mRNA in response to the parasite coincided with a significant down-regulation of mRNA of anti-inflammatory IL-10 irrespective of a chronic *T. gondii* infection of the blood donors (Figure 5B). Finally, TNFa mRNA decreased similarly during *in vitro* cultivation or parasite infection as compared to freshly isolated cells, and this occurred likewise in cells from both, *T. gondii* seropositive and seronegative blood donors (Figure 5C). Thus, *in vitro* infection of monocyte-enriched PBMCs with *T. gondii* induced a pro-inflammatory cytokine milieu with higher *il-12b* gene expression in cells from chronically infected toxoplasmosis patients.

## Discussion

A hallmark of *T. gondii* infections of humans and other intermediate hosts is the parasites' persistence for months to years or even for the hosts' life. Chronic human infection underlies life-threatening reactivated toxoplasmosis in immunocompromised patients (Montoya and Liesenfeld, 2004), recrudescent ocular toxoplasmosis in immunocompetent individuals (Pleyer et al., 2014), and it has been associated with several psychiatric disorders (Sutterland et al., 2015) and behavioral changes (Flegr et al., 2002). Not surprisingly, it is accompanied by potent memory B and T cell responses of immunocompetent humans to the parasite, with the latter ones being particularly important to control the parasite (Canessa et al., 1988; Curiel et al., 1993; Prigione et al., 1995; Montoya et al., 1996). Here, we provide the first evidence that peripheral monocytes from humans with chronic toxoplasmosis differ from those of non-infected controls by their repertoire of cell surface markers and their reactivity to *T. gondii in vitro*. This is of major interest since it suggests a long-term impact of the parasite on cells of the innate immune system which is reminiscent to a form of innate immune memory (Quintin et al., 2012; Lachmandas et al., 2016; Schrum et al., 2018), referred to as trained immunity (Netea et al., 2011). Such reprogramming of monocytes during toxoplasmosis might not only influence the host responses to *T. gondii* but possibly also to heterologous pathogens.

*Ex vivo* analyses revealed that monocytes from humans with chronic toxoplasmosis expressed less CD16 and were less frequently CD62L^+^, but more frequently CD64^+^ than monocytes from sero-negative controls. They showed also a trend toward lower expression of CD62L. This repertoire of cell surface markers is remarkable, since it indicates phenotypic changes during chronic toxoplasmosis which are typical for both, CD16^−^ classical monocytes and CD16^+^ intermediate or non-classical monocytes. Low CD16 and high CD64 expression indeed argue for expansion of CD16^−^ monocytes whereas lower expression of CD62L is indicative for CD16^+^ monocytes (Auffray et al., 2009; Ingersoll et al., 2010; Ziegler-Heitbrock et al., 2010). Consistent with this conclusion is our finding that the distribution of the three major human monocyte subsets, i.e., CD14^+^CD16^−^ classical, CD14^+^CD16^+^ intermediate and CD14^dim^CD16^+^ non-classical monocytes (Ziegler-Heitbrock et al., 2010) did not differ between chronic toxoplasmosis patients and naïve controls. Thus, during chronic human toxoplasmosis monocytes from peripheral blood present a distinct phenotype rather than showing expansion of one of the *bona fide* subpopulations. Peripheral blood monocytes are versatile und dynamic cells (Grage-Griebenow et al., 2001; Auffray et al., 2009) which consecutively develop from rather short-lived classical monocytes to intermediate and finally non-classical monocytes (Patel et al., 2017). The results presented here with monocytes from chronic toxoplasmosis patients highlight the plasticity of human monocytes.

Beside phenotypic differences as observed directly after cell isolation, monocytes from *T. gondii* seropositive humans partially also presented different responses to direct exposure to parasite infection than those from *T. gondii* naïve individuals. Though not specific to the *in vitro* infection, they expressed less CD14 after encountering *T. gondii*, as compared to seronegative humans. Remarkably, they specifically up-regulated HLA-DR,DP,DQ in response to parasite infection and up-regulated IL-12b mRNA more vigorously than cells from seronegative humans. It must be stressed that we did not FAC-sort monocytes before analyzing cytokine transcript levels, and we can therefore ascribe the higher IL-12b expression only to monocyte-enriched PBMCs. After exposure to live parasites, CD16^+^ monocytes and CD1c^+^ DCs are major producers of IL-12 in humans *ex vivo* (Tosh et al., 2016). The contribution of each of these cell populations to the vigorous IL-12b mRNA as observed in monocytes-enriched PBMCs from chronically infected toxoplasmosis patients therefore needs to be further investigated.

We also uncovered several responses to *in vitro* parasite infection by both, monocytes from seropositive and seronegative humans. Thus, CD14^+^CD16^−^ classical monocytes clearly increased with a concomitant decrease of the CD14^+^CD16^+^ and CD14^dim^CD16^+^ subsets, and they generally expressed less CD14 and CD16 than non-infected control cells. Furthermore, CCR2 expression was almost completely down-regulated whereas CD64 expression increased. Finally, IL-10 mRNA levels also clearly decreased in cells from both groups of humans, although this can again only be ascribed to monocyte-enriched PBMCs (see above). Expansion of CD14^+^CD16^−^ monocytes, down-regulation of IL-10 mRNA, and up-regulation of IL-12b mRNA as observed with cells from both groups of humans (albeit significantly higher in those from *T. gondii* seropositive ones; see above) are indicative for an inflammatory cell phenotype. CD14^+^CD16^−^ classical monocytes are considered the human equivalent of murine Ly6c^+^ (Gr1^+^) monocytes (Auffray et al., 2009; Ingersoll et al., 2010; Ziegler-Heitbrock et al., 2010). The latter ones are often referred to as “inflammatory” monocytes although this attribute may be too simplistic (Ziegler-Heitbrock et al., 2010). Although the cytokine profile indeed argues for a pro-inflammatory function, it is interesting to note that CCR2 levels and positivity were strongly decreased in response to parasite infection *in vitro*. This chemokine receptor is among the *bona fide* monocyte subsets restricted to human CD14^+^CD16^−^ or murine Ly6c^+^ classical monocytes. It is required for the emigration of monocytes from the bone marrow into the blood circulation and for the subsequent recruitment to sites of injury or infection (Shi and Pamer, 2011), including acute infections of mice with *T. gondii* (Robben et al., 2005; Dunay et al., 2008). Expansion of CD14^+^CD16^−^ classical monocytes and concomitant down-regulation of CCR2 in response to *T. gondii* thus point toward development of monocytes with a distinct phenotype that do not resemble the three main human subsets. It is reminiscent to a versatile monocyte reprogramming after they encounter site-specific or signal-specific environments (Arnold et al., 2007; Avraham-Davidi et al., 2013).

The functional consequences of monocyte reprogramming during chronic human toxoplasmosis and in response to *in vitro* parasite infection yet remain to be uncovered. Due to the distinct cell phenotypes with characteristics of different monocyte subsets they are also not easy to predict. The increase in positivity or expression of the high affinity IgG receptor (FcγRI, i.e., CD64) nevertheless suggests increased phagocytic activity of monocytes from *T. gondii* seropositive humans and after direct exposure to the parasite *in vitro*. The up-regulation of HLA-DR,DP,DQ as specifically observed in response of monocytes from chronic toxoplasmosis patients to parasite infection *in vitro* additionally argues for increased capacities to present antigens to CD4^+^ T lymphocytes. These findings are remarkable since human monocytes in contrast to their murine counterparts need to phagocytose *T. gondii* in order to subsequently induce cytokine production (Tosh et al., 2016). Finally, the decrease in CD62L^+^ cells and the trend toward reduced CD62L expression levels suggest reduced capacities of monocytes from humans with chronic toxoplasmosis to adhere to endothelial cells and to transmigrate into the surrounding tissue (Rzeniewicz et al., 2015). The severe down-regulation of CCR2 after direct exposure of monocytes from both seropositive and seronegative humans to the parasite might further contribute to a reduced recruitment of human monocytes to sites of infection or injury. However, these assumptions clearly need to be confirmed in humans with chronic toxoplasmosis although this will be a challenging task.

It is generally assumed that humans chronically infected with *T. gondii* harbor a restricted number of tissue cysts predominantly in brain and muscle tissues, though only few studies mostly with AIDS patients have addressed these issues (reviewed in Mcconkey et al., 2013; Wohlfert et al., 2017). Recent findings indicate that bradyzoites within tissue cysts may be more active than previously thought (Watts et al., 2015), and tissue cysts might even rupture occasionally with the majority of parasites being rapidly cleared by the host's immune response. Even on such occasion the inflammatory responses remain however locally confined to the respective sites of parasite reactivation. It is therefore rather unlikely that a limited number of more or less dormant intracellular parasites in brain and muscle tissues induces those changes in phenotypes and reactivity of monocytes from peripheral blood as described here. We instead propose that these changes are signs of a monocyte reprogramming during the acute phase of infection. Human monocytes are readily infected and permissive to rapid tachyzoite division (Channon et al., 2000), and studies in mice show that blood monocytes are critical for parasite dissemination (Courret et al., 2006). Acute infection with *T. gondii* consequently induces a robust innate immune response in humans and their monocytes are readily activated after phagocytosis of live parasites (Sher et al., 2017). Remarkably, accumulating evidence suggests that sensing of distinct pathogen-associated molecular patterns from bacteria, fungi and parasites through pattern recognition receptors can alter functionality of monocytes in the long term (Quintin et al., 2012; Lachmandas et al., 2016; Schrum et al., 2018). This form of innate immune memory, i.e., trained immunity, seems to be regulated by epigenetic mechanisms and metabolic reprogramming of monocytes and their progenitor cells (Quintin et al., 2012; Bekkering et al., 2018; Mitroulis et al., 2018; Schrum et al., 2018). Importantly, the reprogrammed monocytes can respond to secondary stimulation by the same but also by heterologous stimuli with enhanced reactivity. This resembles previous reports on chronically *T. gondii*-infected mice which are more resistant to challenge infections with bacterial pathogens (Ruskin and Remington, 1968; Ruskin et al., 1969; Neal and Knoll, 2014). Thus, the altered phenotypes and reactivity of human monocytes as reported herein suggest that innate immune training may also operate during infections with *T. gondii* but this awaits future confirmation.

## Ethics Statement

This study was carried out in accordance with the recommendations of Ethics commission of the University Medical School Göttingen with written informed consent from all subjects. The protocol was approved by the Ethics commission of the University Medical School Göttingen (Project numbers 28/3/08 and 1/3/13).

## Author Contributions

CL conceived the study and the experimental setup, prepared the figures, and wrote the manuscript. HE conducted the experiments and analyzed the data. Both authors read and approved the final version of the manuscript.

### Conflict of Interest Statement

The authors declare that the research was conducted in the absence of any commercial or financial relationships that could be construed as a potential conflict of interest.
