# Supplementary material for: Long-Term Impact of Toxoplasma gondii Infection on Human Monocytes
Source: Front Cell Infect Microbiol. 2019 Jun 28;9:235. doi: 10.3389/fcimb.2019.00235 (PMC6611340; doi:10.3389/fcimb.2019.00235)
Supplement: Supplementary file 1 [file Data_Sheet_1.docx]

***Supplementary Material***

1. **Supplementary Figures and Tables**
   1. **Supplementary Table**

**Supplementary Table 1:** Serology of *T. gondii*-positive blood donors**.**

| Total Ig Competition | IgG [IU/ml] | IgM [Index values] | IgA | IgG avidity |
| --- | --- | --- | --- | --- |
| 0.03* | 55 | < 0.65** | negative | nd |
| 0.06 | 24 | < 0.65 | negative | nd |
| 0.00 | > 300 | 1.95 | negative | high |
| 0.03 | 63 | 0.94 | negative | high |
| 0.01 | 267 | < 0.65 | negative | nd |

*Data are inversely proportional to *T. gondii* IgG/IgM; values < 1.6 are considered positive.

**Index values > 0.65 are considered positive.

Nd: not determined.

- 1. **Supplementary Figures**


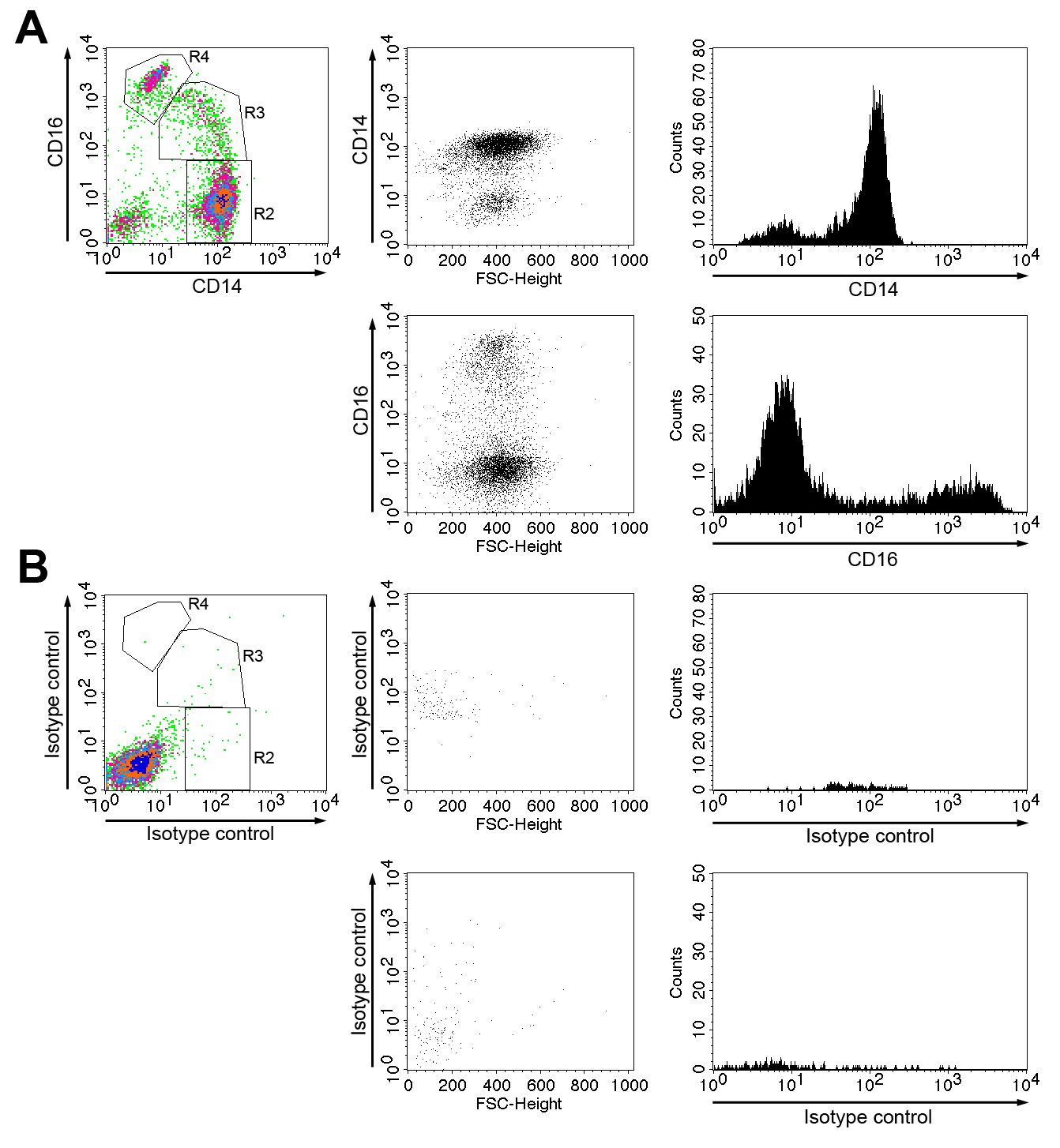


**Supplementary Figure 1.** Representative FACS analysis of CD14 and CD16 expression levels on monocytes from *T. gondii* sero-positive or sero-negative blood donors. (A) Freshly isolated monocyte-enriched PBMCs were fluorescently labelled with anti-CD14 and anti-CD16 antibodies and FSC/SSC-gated monocytes were classified as CD14^+^CD16^-^ (R2), CD14^+^CD16^+^ (R3) and CD14^dim^CD16^+^ (R4) subpopulations (upper left panel; see also Fig. 1A). Expression levels of CD14 (upper panel) and CD16 (lower panel) were then determined for R2+R3+R4-gated cells. (B) Control staining with isotype control antibodies was done in parallel.


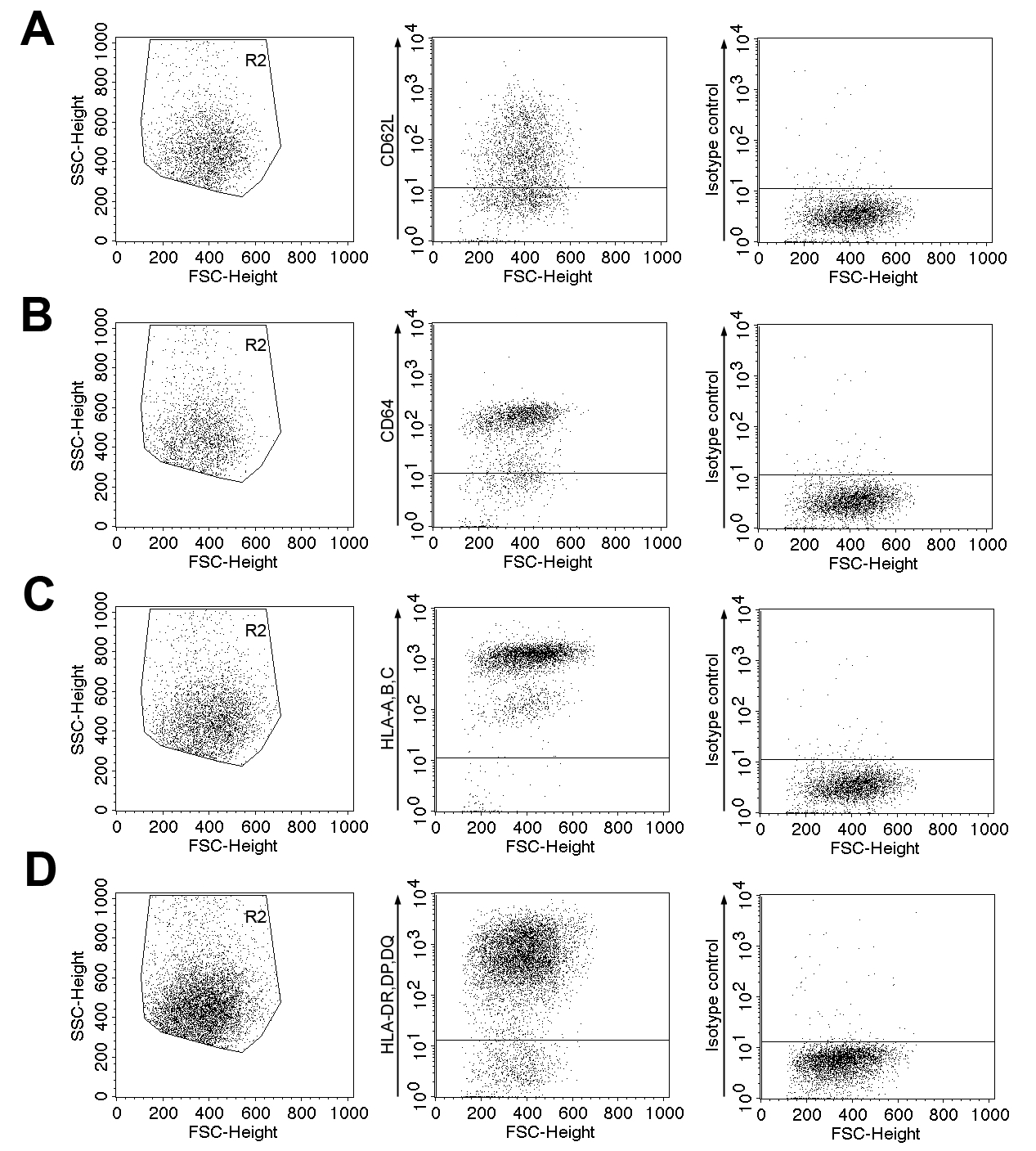


**Supplementary Figure 2.** Representative FACS analysis of surface markers on monocytes from *T. gondii* sero-positive or sero-negative blood donors. Freshly isolated monocyte-enriched PBMCs were fluorescently labelled using anti-CD14, or antibodies directed against CD62L, CD64, HLA-A,B,C and HLA-DR,DP,DQ (A-D; middle panels), or isotype control antibodies (A-D; right panels). CD14^+/dim^ monocytes were identified among FSC/SSC-analyzed cells (R2 in left panels; see also Fig. 2A), and were analyzed for expression of surface markers as indicated. Positive cells were identified by expression above background staining with isotype control antibodies. Expression levels of the surface markers were determined for R2-gated cells.
